# Supplementary material for: Identification of photoperiod-induced specific miRNAs in the adrenal glands of Sunite sheep (Ovis aries)
Source: Front Vet Sci. 2022 Jul 22;9:888207. doi: 10.3389/fvets.2022.888207 (PMC9354845; doi:10.3389/fvets.2022.888207)
Supplement: Supplementary file 1 [file Data_Sheet_1.ZIP › Supplementary file/miRDeep2 file.zip!/miRDeep2 file/Novel_Structure/LP42A2/Novel_612.pdf]

Provisional ID	:	NC_019481.2_35367
Score total	:	3
Score for star read(s)	:	-1.3
Score for read counts	:	0
Score for mfe	:	2.7
Score for randfold	:	1.6
Score for cons. seed	:	
Total read count	:	46
Mature read count	:	45
Loop read count	:	0
Star read count	:	1

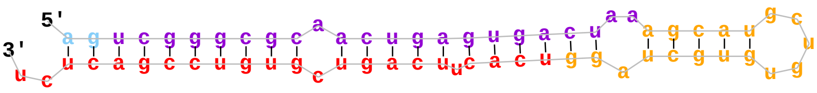

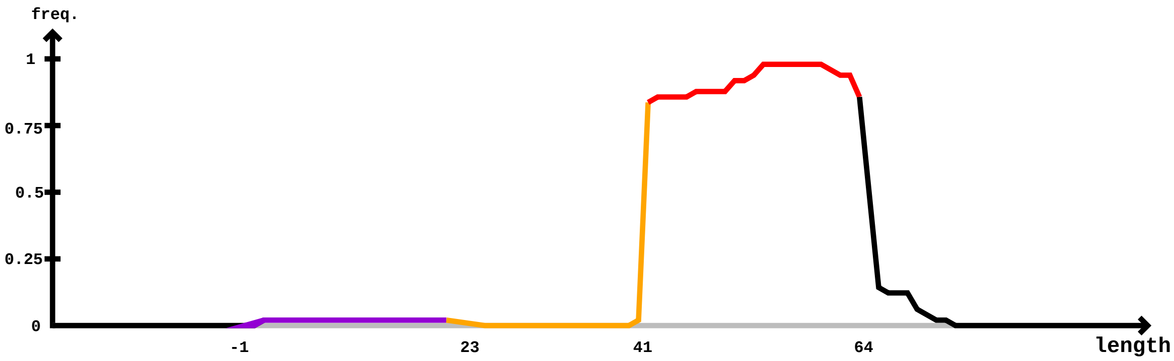

Star **Mature**

```
5'- guccaugggggugcaaagagucgggcgcaacugagugacuaaagcaugcugugugcuaggucacuucagucguguccgacucuuugcaacccuuuggacuguagcccaccag   -3'   obs
    guccaugggggugcaaagagucgggcgcaacugagugacuaaagcaugcugugugcuaggucacuucagucguguccgacucuuugcaacccuuuggacuguagcccaccag         exp
    (((((.((((.((((((((((((((((.((((((((((..(((((.....))))).)))))).))))).)))))))))))))))).)))).))))).............   reads   mm   sample
    ....................ucgggcgcaacugagugacuaa......................................................................   1   0   seq
    ...........................................................gucacuucagucguguccgacucu.............................   1   0   seq
    ............................................................ucacuucagucguguccga.................................   1   0   seq
    ............................................................ucacuucagucguguccgaU................................   1   1   seq
    ............................................................ucacuucagucguguccgacuc..............................   2   0   seq
    ............................................................ucaAuucagucguguccgacuc..............................   2   1   seq
    ............................................................ucacuucagucguguccgacucA.............................   10  1   seq
    ............................................................ucacuucagucguguccgacucG.............................   2   1   seq
    ............................................................uAacuucagucguguccgacucu.............................   1   1   seq
    ............................................................ucaAuucagucguguccgacucu.............................   4   1   seq
    ............................................................ucaGuucagucguguccgacucu.............................   3   1   seq
    ............................................................ucacuucagucguguccgacucC.............................   12  1   seq
    ............................................................ucacuucagucguguccgacucCu............................   1   1   seq
    ............................................................ucacuucagucguguccgacucuuu...........................   1   0   seq
    .............................................................cacuucGgucguguccgacucu.............................   1   1   seq
    .................................................................ucagucguguccgacucuuugUa........................   1   1   seq
    .....................................................................ucguguccgacucuuugUa........................   1   1   seq
    .....................................................................ucUuguccgacucuuugca........................   1   1   seq
    .......................................................................guguccgacucuuugcaac......................   1   0   seq
    ........................................................................uguAcgacucuuugcaa.......................   1   1   seq
    ........................................................................uguccgacucuuugcaGccc....................   1   1   seq
```
